# A Versatile System for USER Cloning-Based Assembly of Expression Vectors for Mammalian Cell Engineering

**DOI:** 10.1371/journal.pone.0096693

**Published:** 2014-05-30

**Authors:** Anne Mathilde Lund, Helene Faustrup Kildegaard, Maja Borup Kjær Petersen, Julie Rank, Bjarne Gram Hansen, Mikael Rørdam Andersen, Uffe Hasbro Mortensen

**Affiliations:** 1 Department of Systems Biology, Technical University of Denmark, Kgs. Lyngby, Denmark; 2 The Novo Nordisk Foundation Center for Biosustainability, Technical University of Denmark, Hørsholm, Denmark; Imperial College London, United Kingdom

## Abstract

A new versatile mammalian vector system for protein production, cell biology analyses, and cell factory engineering was developed. The vector system applies the ligation-free uracil-excision based technique – USER cloning – to rapidly construct mammalian expression vectors of multiple DNA fragments and with maximum flexibility, both for choice of vector backbone and cargo. The vector system includes a set of basic vectors and a toolbox containing a multitude of DNA building blocks including promoters, terminators, selectable marker- and reporter genes, and sequences encoding an internal ribosome entry site, cellular localization signals and epitope- and purification tags. Building blocks in the toolbox can be easily combined as they contain defined and tested Flexible Assembly Sequence Tags, FASTs. USER cloning with FASTs allows rapid swaps of gene, promoter or selection marker in existing plasmids and simple construction of vectors encoding proteins, which are fused to fluorescence-, purification-, localization-, or epitope tags. The mammalian expression vector assembly platform currently allows for the assembly of up to seven fragments in a single cloning step with correct directionality and with a cloning efficiency above 90%. The functionality of basic vectors for FAST assembly was tested and validated by transient expression of fluorescent model proteins in CHO, U-2-OS and HEK293 cell lines. In this test, we included many of the most common vector elements for heterologous gene expression in mammalian cells, in addition the system is fully extendable by other users. The vector system is designed to facilitate high-throughput genome-scale studies of mammalian cells, such as the newly sequenced CHO cell lines, through the ability to rapidly generate high-fidelity assembly of customizable gene expression vectors.

## Introduction

The medical use of therapeutic proteins is in rapid growth and their full potential in health care is vast. More than 200 approved biopharmaceuticals are already on the market, with the most rapidly developing market being monoclonal antibody (mAb)-based products [1]. Accordingly, the interest in construction and development of mammalian cell factories for production of therapeutic proteins is increasing. This interest has been further stimulated by the recent publication of the first genome drafts of CHO cell lines [2]–[3], the primary host for expression of human proteins in general, and antibodies in particular [1]. These publications open new avenues of genetic engineering of these important cell factories [4].

Vector systems for these mammalian cell lines have so far mainly focused on efficient expression of recombinant proteins. This has primarily been achieved by relatively simple and inflexible vector systems containing a strong promoter (often viral) followed by a multiple cloning site (MCS), a terminator, and a selectable marker. The vectors are thus typically assembled by conventional methods based on the use of restriction endonucleases and ligases. Vector construction will therefore often be hampered by a number of limitations such as few restriction enzyme cut sites or cloning method incompatibilities between plasmid and desired DNA inserts. Moreover, it is next to impossible to assemble more than two DNA fragments in a single restriction/ligation step. For these reasons, restriction enzyme and/or ligase-independent techniques, e.g. In-Fusion cloning, Gibson assembly and USER cloning, are getting increasingly popular in many other cell systems [5]. These techniques allow seamless and directed assembly of vector fragments and inserts that are enabled by single stranded DNA overhangs [6]–[12]. In the case of the USER cloning method, the overhangs are generated by substituting a single deoxy-thymidine nucleotide with a deoxy-uridine nucleotide in the 5′ end of each primer designed to amplify the desired genetic target. Subsequently, the resulting PCR DNA fragment is treated with the USER enzyme-mix (Uracil DNA glycosidase and DNA glycosylase-lyase endo VIII) resulting in formation of unique 3′ single stranded overhangs [9]–[12]. Importantly, *in vivo* fusions of DNA fragments are so efficient that several fragments can be combined in a single round of cloning. The method is PCR-based and can therefore easily be used to introduce modifications, e.g. point mutations and linkers, into a DNA fragment during the assembly process [10]–[13]. Furthermore, the method is very suited for high-throughput setups [8], [12].

For efficient high-throughput cloning and advanced genetic engineering, it is also of importance to have a modular vector system, especially if one wishes to have flexibility of the inserted components. Possibly the best known system of this type is the BioBrick standard, developed by the iGEM Foundation (www.igem.org). It provides a modular assembly standard, and is non-commercial, but is limited by BioBricks requiring restriction enzymes for plasmid integration. Biobricks are thus difficult to apply to mammalian systems where genes are typically very long genes and contain multiple bacterial restriction enzyme cut sites. Combining several modules also becomes a multiple step operation.

In the present study, we have developed and characterized a non-commercial comprehensive vector expression platform for mammalian cell engineering based on the DNA ligase-free uracil-excision based USER cloning method [9]. The platform contains a basic set of fixed shuttle vectors (pBASE) for high-throughput cloning of specific genes of interest (GOI). In addition, it contains a flexible multipurpose DNA fragment toolbox containing sequence building blocks that are equipped with Flexible Assembly Sequence Tags, FASTs, allowing them to be easily fused by USER fusion [11]. Using FASTs, the building blocks can be used for rapid construction of *E. coli* shuttle vectors with different mammalian selection markers where GOIs can be equipped with a variety of promoters and terminators or combined with sequence encoding internal ribosome entry sites for bicistronic gene expression. Moreover, the toolbox contains FAST building blocks encoding reporter proteins and cellular localization sequences, as well as purification and epitope tags. To demonstrate the potential of our toolbox, we have successfully assembled vectors composed by up to seven building blocks in a single cloning step and provided proof of functionality in mammalian hosts. For example, we have made vectors for protein secretion and for bicistronic gene expression, as well as vectors encoding chimeric fluorescent proteins, which were successfully used as cytological markers in U-2-OS cells.

## Materials and Methods

### Strains, cell cultures and media

All standard cloning and plasmid propagation was performed in *Escherichia coli* strain DH5α, which was grown in standard Luria Broth (LB) medium supplemented with 100 µg/ml ampicillin.

Human U-2-OS osteosarcoma cells and HEK293 cells both obtained from ATCC were grown in Dulbecco's modified Eagle's medium (Lonza, Vervier, Belgium) supplemented with 10% (v/v) FBS (Lonza), 100 µg/ml streptomycin and 100 U/ml penicillin (Lonza). CHO-S suspension cells obtained from Life Technologies were grown in CD CHO medium (Gibco, Life Technologies) supplemented with 8 mM L-Glutamine (Gibco, Life Technologies) and 1∶500 Anti-Clumping Agent (Gibco, Life Technologies).

### Plasmids and primers

All plasmids used as PCR templates in this study are listed in Table 1. The pIRES-DHFR plasmid is a modified version of the IRES domain of the plasmid pIRES (Clontech, Palo Alto, CA, USA), which was linked to the dihydrofolate reductase (DHFR) gene in house. Oligonucleotides for PCR are listed in Table S1 and oligonucleotides for DNA hybridization of building blocks are listed in Table S2. Plasmids generated in this study are found in Table S3.

**Table 1:** Plasmids applied as PCR templates in this study.

*Plasmid*	*Functional elements*	*Source*
pcDNA3.1(+)	pCMV, pSV40, SV40 pA, BGH pA, and NeoR	Invitrogen
pcDNA3.1/Hygro(+)	HygR	Invitrogen
pFLAG-CMV	hGH pA, FLAG-tag	Sigma-Aldrich
pSUPERIOR.puro	pPGK	Oligoengine
peGFP-1	eGFP	Clontech
peYFP-C1	eYFP	Clontech
peCFP-C1	eCFP	Clontech
pmCherry-N1	mCherry	Clontech
pGEM-4Z/PLAP489	SEAP	[14]
pU0020	AmpR, pUC19 replication origin	[13]
pIRES-DHFR	IRES, DHFR	This study

AmpR: ampicillin resistance gene; BGH: bovine growth hormone; CMV: cytomegalovirus; DHFR, dihydrofolate reductase; eCFP: enhanced cyan fluorescent protein; eGFP: enhanced green fluorescent protein; eYFP: enhanced yellow fluorescent protein; hGH: human growth hormone; HygR: hygromycin resistance gene; IRES: internal ribosomal entry site; mCherry: monomeric Cherry fluorescent protein; NeoR: neomycin resistance gene; pA: polyadenylation signal; PGK: phosphoglycerate kinase-1; SEAP: secreted alkaline phosphatase; SV40: Simian virus 40.

### Construction of DNA building blocks

DNA building blocks were amplified by PCR using the proofreading polymerase *Pfu*X7 [15]. All primers for amplification of the DNA building blocks were designed based on commercially available vectors (Table 1). Furthermore, each primer was extended by a specific FAST at the 5′-end (Table 2). PCRs were performed in 35 PCR cycles in a final volume of 50 µl with addition of 1% MgCl_2_ (New England Biolabs, Ipwich, MA, USA). Targeting signals (TSs) of 9–25 bp were added by inclusion of the appropriate sequence in the primer for the PCR amplification of the GOI as an extension between the FAST and the annealing primer. TSs between 25–100 bp were made by DNA hybridization of two complementary single stranded oligonucleotides. DNA hybridization was performed in Milli-Q water in a final volume of 80 µl with a concentration of 50 µM of each complementary oligo. The mixture was incubated for 5 min at 98°C and left at room temperature overnight before storage at −20°C. For TSs above 100 bp, Fusion PCR (overlapping PCR) were performed on synthetic oligos covering the complete length in first 5 cycles with annealing at 54°C, followed by 30 cycles with annealing at 66°C.

**Table 2:** Standardized USER fusion Flexible Assembly Sequence Tags (FASTs).

Name	Color code^a^	Nucleotide 5′–3′	FW primer tail 5′–3′	RV primer tail 5′–3′
FAST1	Green	ACGTCGCT	ACGTCGCU	AGCGACGU
FAST2	Light blue	AGTGCGAT	AGTGCGAU	ATCGCACU
FAST3	Dark blue	AGCGCTGGT	AGCGCTGGU	ACCAGCGCU
FAST4	Yellow	ACTATGCCT	ACTATGCCU	AGGCATAGU
FAST5	Purple	ACACAGTCT	ACACAGTCU	AGACTGTGU
FAST6	Pink	ACTTGCGT	ACTTGCGU	ACGCAAGU
FAST7	Red	ATTAAGCT	ATTAAGCU	AGCTTAAU

FW: forward; RV: reverse.

a All color codes match Figure 3.

PCR of building blocks encoding vector backbones were subjected to *Dpn*I (New England Biolabs) digestion (20 U, 37°C, 1 h), heat inactivation (80°C for 20 min) and purified by gel. All PCR products were isolated by agarose gel separation and purified using illustra GFX™ PCR DNA and Gel Band Purification Kit (GE Healthcare, Buckinghamshire, United Kingdom).

### Construction of expression vectors

USER cloning and USER fusion was performed as previously described [9], [11] with minor modifications: Vector backbones holding a PacI/Nt.BbvCI USER cloning compatible cassette were digested as previously described [13]. 1 µl of USER enzyme mix, 0.5 µl NEB Buffer 4, 0.5 µl BSA x10 (all purchased from New England Biolabs), and 0.1 pmol digested vector backbone were mixed in a 0.2 ml PCR tube. If vector backbone was amplified by PCR, 1 µl purified DNA element was added. Finally, 7 µl of purified DNA elements were mixed to a total reaction volume of 10 µl. If more than one DNA element was to be inserted, all elements were added in equal volumes. The reaction mixture was incubated for 40 min at 37°C, followed by 30 min at 25°C. Subsequently the 10 µl reaction mixture was used directly to transform chemically competent *E. coli* DH5α cells. Transformants were selected in Luria Broth (LB) medium supplemented with 100 µg/ml ampicillin. Three transformants were picked randomly for each construct and validated by DNA sequencing (Star SEQ, Mainz, Germany). For each construct, a validated expression vector was purified by Plasmid Plus Maxi kit (Qiagen, Hilden, Germany) following the manufacturer's instruction and dilution to a concentration of 1 µg/µl DNA in MilliQ water.

### Cell cultivation and transfection

All cells were cultivated in a humidified incubator at 37°C with 5% CO_2_. CHO-S cells were expanded in Erlenmeyer cell culture flasks (Corning, Sigma-Aldrich) and experiments were performed in uncoated 6 well plates (Falcon, BD Biosciences). Adherent cell lines were passaged by exposure to 0.05% trypsin-EDTA (Lonza), when fully confluent and were seeded to a confluence of 20% the day prior to transfection. CHO-S cells were transfected (Day 0) in a Nucleofector 2b using the Amaxa Cell Line Nucleofector Kit V (Lonza). A total of 2•10^6^ cells were transfected with 2 µg plasmid. Plasmid transfections of adherent cells were performed using X-tremeGENE HP (Roche, Basel, Switzerland) in Gibco Opti-MEM (Life Technologies, Paisley, United Kingdom) medium.

### Fluorescence microscopy

The day after transfection (Day 1) of CHO-S cells, 25,000 cells from each sample were stained with one drop of NucBlue Live Cell Stain (Hoechst33342) for 20 min before analysis using a Celigo Imaging Cell Cytometer (Brooks Automation).

U-2-OS cells were grown on coverslips (VWR BDH Prolabo, Poole, United Kingdom) in 6-well plates (2 coverslips/well). The cells on coverslips were fixed in 4% formaldehyde solution (VWR) for 12 min at room temperature. Subsequently, the coverslips were washed in 1x PBS (Lonza) twice and stored in PBS at 4°C in darkness. Prior to microscopy, the coverslips were mounted onto glass slides with DAPI-containing mounting medium (Vector Laboratories Inc., Burlingame, CA, USA) and preserved with clear nail polish. Confocal laser scanning microscopy was performed for visualization of fluorescent proteins on a Zeiss LSM 780 confocal with an Axio Observer with 63x/1.40 oil DIC Plan-Apochromat. Images were acquired through a Zeiss Zen 2010 digital camera.

### Measurement of secreted eGFP

Transfected HEK293 cell were grown in p60 dishes (Nunc), and 500 µl of medium extract was sampled in triplicates approximately 72, 96, and 120 hours after transfection. The samples were centrifuged for 5 min at 12.000 g and the supernatant collected. 100 µl supernatant was used for quantification of fluorescence intensity determined in flat-bottomed micro-titer plates (Nunc) on a Synergy 2 Microplate Reader (BioTek Instruments Inc., Burlington, VT, USA) for emission of a wavelength of 485 nm within a range of 20 nm.

### SEAP assay

SEAP activity was measured as described by Durocher et al. [16]. Transiently transfected HEK293 cells were cultured in p100 dishes (Nunc) and culture medium was harvested approximately 48 hours after transfection. The samples were centrifuged for 5 min at 12.000 g and the supernatant was collected. To inhibit endogenous phosphatase activities, samples were heat-inactivated at 60°C for 30 minutes. 50 µL samples were transferred to a 96-well microtiter plate and mixed with an equal volume of SEAP assay solution containing 20 mM para nitrophenyl phosphate (pNPP) (Amresco, Solon, OH, USA), 1 mM MgCl_2_ (Sigma-Aldrich, St. Louis, MO, USA), and 1 M diethanolamine (Sigma-Aldrich), adjusted to pH 9.8. Absorbance was read at 410 nm in 1-minute intervals using a Synergy 2 Microplate Reader at 37°C to determine pNPP hydrolysis rates. The level of SEAP was calculated based on the specific activity of SEAP being 2000 mU/µg protein where 1 mU corresponds to an increase in light absorbance at 410 nm of 0.04 units/min.

## Results and Discussion

### Construction of a basic set of mammalian expression vectors

**Figure 1:**
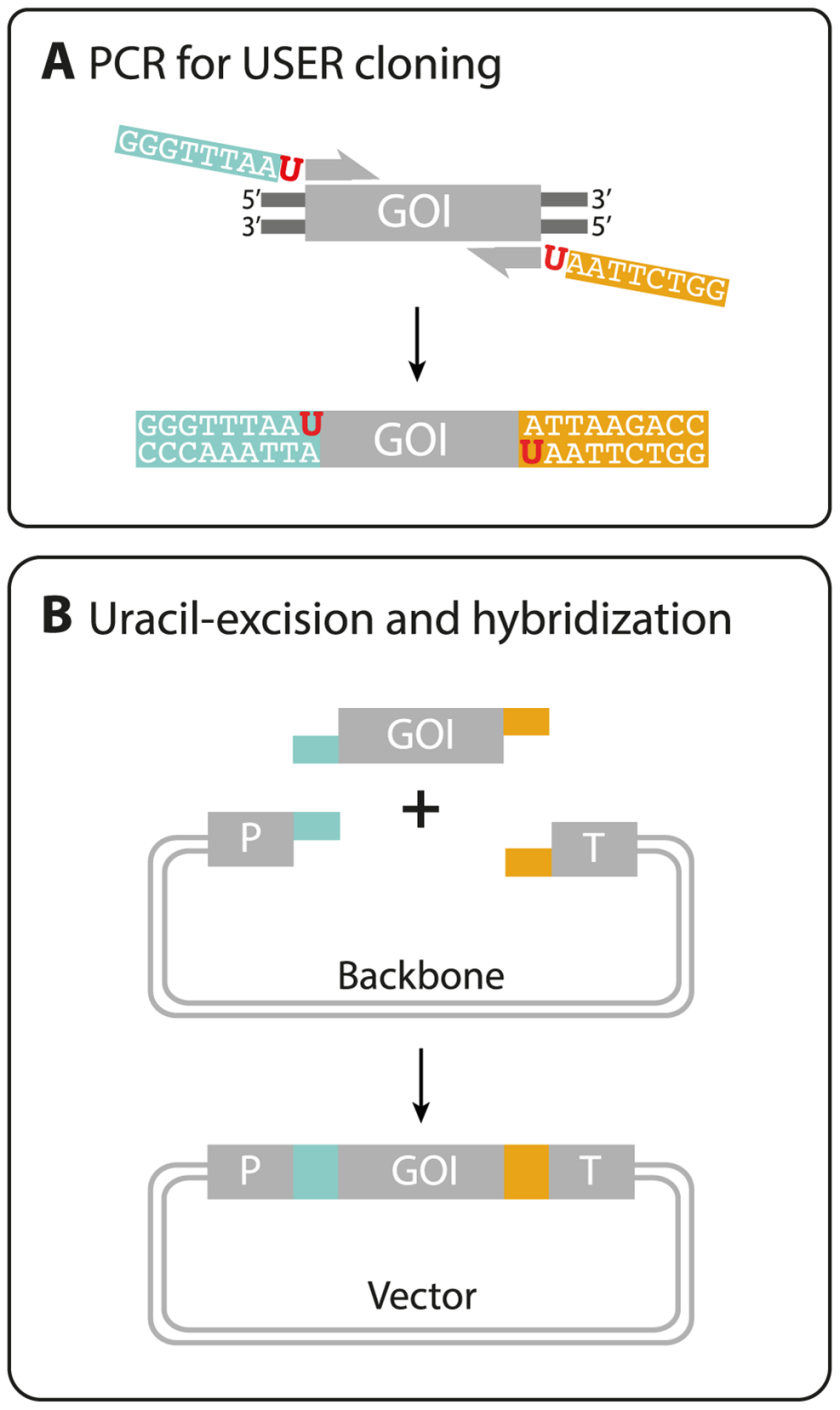
Integration of a GOI into the pBASE backbone vector. (**A**) PCR amplification of the GOI using uracil-containing primers and a uracil compatible DNA polymerase (e.g. PfuX7). (**B**) The PCR fragment of B and a vector backbone fragment obtained by PacI/Nt.BbvCI digest (see Figure S1) is mixed. Addition of the USER™ enzyme mix catalyzes uracil-excision and generates compatible overhangs on the PCR fragment for directed vector assembly. Subsequently, the cloning mix is directly transformed into E. coli cells, where the fragments are ligated together.

**Figure 2:**
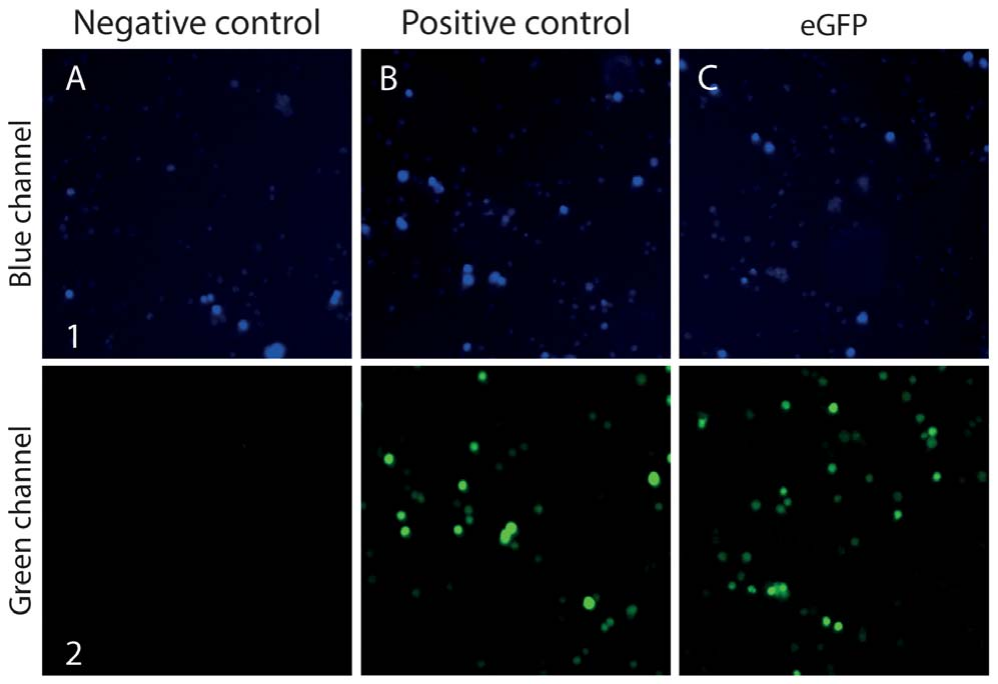
Imaging Cell Cytometry of live CHO-S cells transfected with pBASE-vector. Celigo Imaging Cell Cytometry of live CHO-S cells one day after transfection with (**A**) pC1_ccdB as negative control, (**B**) pFAST1-eGFP as positive control, and (**C**) pBASE2-eGFP as proof of concept for eGFP insertion into the PacI/Nt.BvCI USER cassette of the pBASE2 vector. (**A1**–**C1)** cells stained with Hoechst33342. (**A2**–**C2**) cytometry of cells with a filter for eGFP fluorescence.

We have created a simple, rapid and robust vector system for inserting a GOI into any of six *E. coli* based vector backbones, pBASE1-6. In each case, the GOI is inserted into a PacI/Nt.BvCI USER cassette [13] flanked by a promoter and terminator by USER cloning, see Figure 1 and Figure S1. For mammalian gene expression, the vectors contain combinations of hygromycin (HygR) and neomycin (NeoR) selectable markers; SV40, PGK, and CMV promoters; and SV40 and BGH terminators, see Table S3. The functionality of the vector design was verified by inserting the gene encoding enhanced green fluorescent protein (eGFP) into pBASE2 for proof of concept. Similar to our previous experience using the PacI/Nt.BvCI cassette for cloning [13], *E. coli* transformants were readily obtained (>200 colonies) and identical pBASE2-eGFP vectors were isolated from three of these colonies. One of the isolated pBASE2-eGFP plasmids was transfected into CHO-S cells for further analysis. A strong green fluorescent signal was detected in the cells (Figure 2). In contrast, CHO-S cells transfected with control plasmid did not show increased fluorescence. Thus, the vector system allows integration of a GOI into a fixed vector in a simple USER fusion reaction.

### Design of a flexible multipurpose DNA fragment toolbox

**Figure 3:**
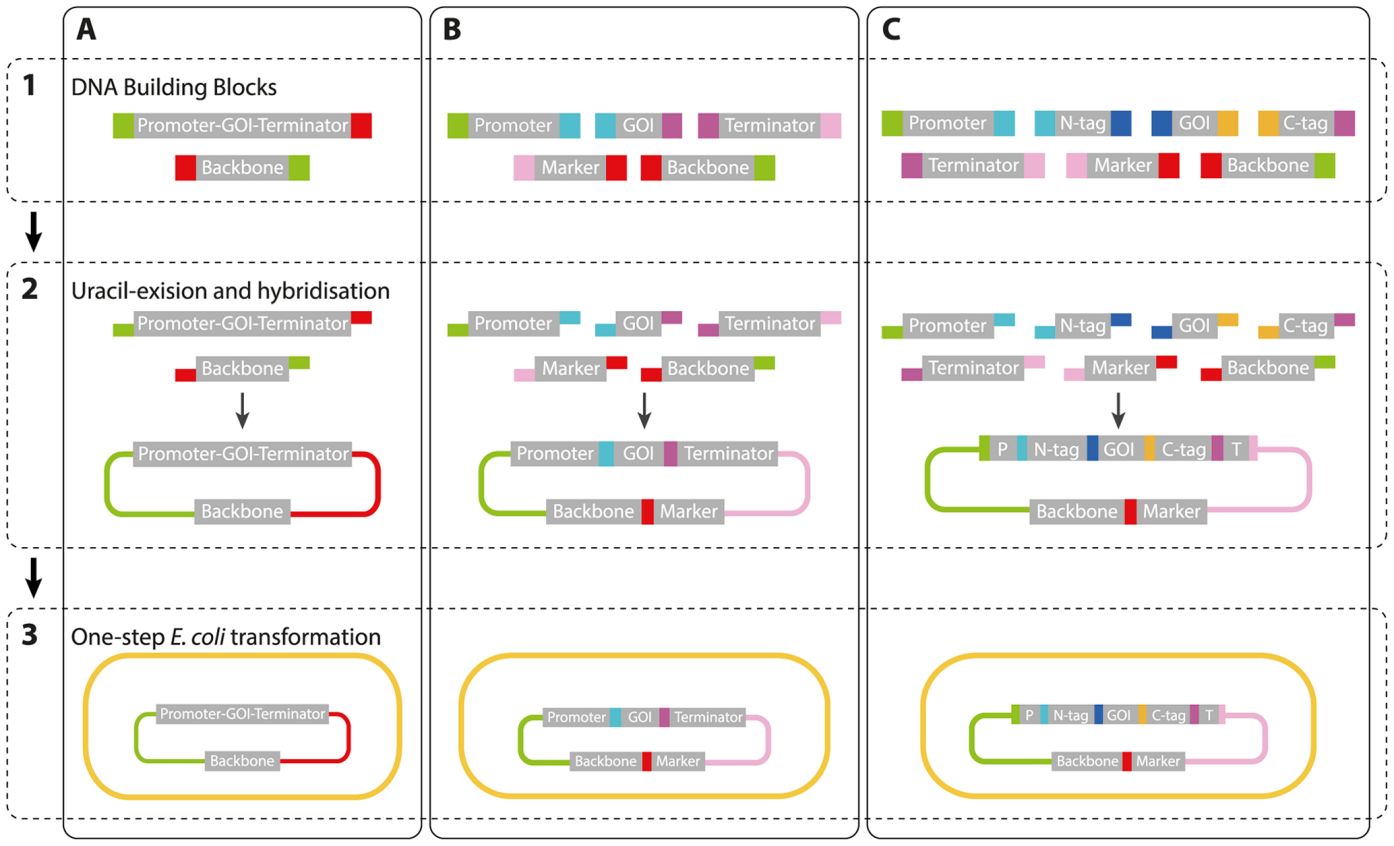
FAST-mediated vector assembly. Construction of vector types (**A**–**C**) requires the same three steps: **1:** Preparation of building blocks with appropriate FASTs by PCR or annealing of complementary oligonucleotides. **2:** USER fusion and hybridization: the USER enzyme and all building blocks are mixed in one reaction. **3:** *E. coli* transformation with the USER cloning reaction mix. (**A**) Insertion of a promoter-GOI-terminator expression cassette in an *E. coli* vector backbone. (**B**) Assembly illustrated with five elements: the expression cassette as three building blocks, an interchangeable selection marker, and a vector backbone. (**C**) Similar to (**B**), but with seven building blocks including C- and N-terminal tags. The N- and C-terminal tag can either be a reporter, a fusion protein, a localization sequence or an epitope tag. GOI: gene of interest; N-tag: N-terminal sequence tag; C-tag: C-terminal sequence tag; P, promoter; and T, terminator.

In many cases, it is necessary to combine multiple DNA elements in a single vector to achieve the desired sequence. This task can easily be achieved with USER fusion where assembly of up to four fragments has previously been demonstrated [10]–[13]. Exploiting this possibility, we have designed a DNA fragment toolbox where the individual DNA building blocks can be combined in a flexible manner for the construction of a multitude of vectors. The individual building blocks in the toolbox have been made either by PCR or by simple annealing of complementary oligonucleotides. The vectors are assembled by combining the individual building blocks by USER fusion via FASTs (Figure 3, Table 2). The FASTs noted in Table 2 have been tested and verified for functionality, but can in principle be changed to suit the needs of the researcher. Each building block is capped with two defined FASTs at either end to allow for directionally controlled fusions to other building blocks in the toolbox. Vector assembly does not require USER cassettes as the vector backbone is generated by PCR. In the present version of our toolbox we have designed seven FASTs, which can mediate assembly of up to seven individual building blocks. Hence, the toolbox supports vector construction with different levels of complexity, see below.

Firstly, in the simplest scenario, it is possible to swap building blocks in the constructs that are based on the basic vector set described above, e.g. if another selectable marker or vector backbone is desirable (Figure 3A). Secondly, a GOI can be combined with a new set of building blocks to form vectors with a configuration similar to those in the basic expression vector set, but with compositions of promoters, terminators, markers and vector backbone, which are not included in the set (Figure 3B). The building blocks harboring the promoter, terminator and marker have fixed positions in the vector relative to the backbone, and for that reason they are always equipped with the same FASTs. In this way there is full flexibility to choose between the three promoters, two terminators, three mammalian markers and two vector backbones that are currently in the toolbox. In total this amounts to 36 vector combinations; a number that will expand as new promoters and terminators are added to the toolbox. The toolbox is not limited to these components, as one can freely add more components to suit specific projects. The only requirement is the addition of the defined FASTs to PCR amplification primers. Thirdly, the toolbox contains building blocks that allow for the construction of expression vectors where the GOI is fused to one or more sequences encoding relevant sorting signals, reporter proteins, and purification/epitope tags (Figure 3C). Currently, the toolbox contain building blocks encoding a ER signal peptide; ER retention- and Golgi retention signals; mitochondrial-, nuclear-, peroxisomal-, and plasma membrane localization signals; reporter proteins including eGFP, eCFP, eYFP, mCherry and secreted alkaline phosphatase (SEAP); and the His6, FLAG and cMyc tags, see Table 3. Building blocks coding for protein are fused with FASTs encoding three amino acid residues, which serve as linkers between the two protein-based components (Table 2). For each of these building blocks, variants exist with different FASTs (Figure S2). As a result, the composition of the FASTs and the relative positioning of building blocks are flexible. This part of the toolbox allows any GOI encoded protein to be fused, N- or C- terminally, with any of the tags for localization, purification and visualization mentioned above. Lastly, vectors supporting mammalian bicistronic gene expression can be constructed as one of the building blocks in the toolbox is an internal ribosome entry site, IRES. This mode of gene expression is desirable if stoichiometric transcription levels of the individual genes are required.

**Table 3:** Elements included in the pBASE and pFAST vector platforms.

*Category*	*Element*	*Template*	*Reference/Source*
Promoters	CMV	pcDNA3.1(+)	Invitrogen
	PGK	pSUPERIOR.puro	Oligoengine
	SV40	pcDNA3.1(+)	Invitrogen
Terminators	BGH pA	pcDNA3.1(+)	Invitrogen
	SV40 pA	pcDNA3.1(+)	Invitrogen
	hGH pA	pFLAG-CMV	Sigma
Marker cassettes	HygR	pcDNA3.1/Hygro(+)	Invitrogen
	NeoR	pcDNA3.1(+)	Invitrogen
	DHFR	pIRES-DHFR	In house/GenBank: BC005796
Targeting signals	NLS (nuclear)	Synthetic	[17]
	PTS1 (peroxisomal)	Synthetic	[18]
	c-Ha-ras (plasma membrane)	Synthetic	[19]–[20]
	COX-VIII (mitochondrial)	Synthetic	[21]
	CRT (ER)	Synthetic	[22]
	KDEL (ER retention signal)	Synthetic	[23]
	GalNacT1 (*medial*-Golgi)	Synthetic	[24]
	β-1,4 GT (*trans*-Golgi)	Synthetic	[25]
	α-2,6 ST (TGN)	Synthetic	[26]–[27]
Secretion signals	hIFN-γ	Synthetic	[28]–[29]
Proteins	eGFP	peGFP-1	Clontech
	eYFP	peYFP-C1	Clontech
	eCFP	peCFP-C1	Clontech
	mCherry	pmCherry-N1	Clontech
	SEAP	pGEM-4Z/PLAP489	[14]
Other elements	IRES	pIRES-DHFR	In house
	Vector backbone	pU0002	[13]
	His-tag	Synthetic	[30]
	FLAG-tag	Synthetic	Invitrogen
	c-Myc-tag	Synthetic	Invitrogen

α-2,6 ST: N-terminal targeting signal of beta-galactoside alpha-2,6-sialyltransferase; β-1,4 GT: N-terminal targeting signal of beta-1,4-galactosyltransferase; c-Ha-ras: C-terminal targeting signal of c-Ha-ras p21 protein; COX-VIII: N-terminal targeting signal of cytochrome c oxidase subunit VIII; CRT: N-terminal targeting signal of calreticulin; ER: endoplasmic reticulum; GalNAcT1: N-terminal targeting signal of N-acetylgalactosaminyltransferase; hIFN-γ: human Interferon-gamma; NLS: C-terminal nuclear localization sequence; PTS1: C-terminal peroxisomal target signal 1; TGN: *trans-*Golgi network.

### Efficiency of the versatile FAST vector assembly system

In order to benchmark the assembly efficiency of the FAST system and the functionality of the assembled vectors, a comprehensive set of mammalian vectors based on different numbers of PCR derived building blocks (five, six or seven) were constructed (Table 4, Table S1). By testing vectors with 5–7 blocks, all FASTs are also validated for functionality. Nine, six, and eleven vectors were successfully made by fusing five, six and seven building blocks, respectively. Importantly, in all these 26 experiments, *E. coli* transformants were easily obtained. However, we noted that the number of colonies decreased as the number of building blocks was increased; hence, the lowest number of transformants, was obtained for a vector that required fusion of seven building blocks (Table 4). For all experiments, three randomly picked colonies were analyzed for the quality of vector assembly. Like the number of transformants, the fusion fidelity also decreased as the number of building blocks was increased. Nevertheless, in the 11 attempts to fuse seven building blocks, 93% of the 33 tested colonies contained a correctly assembled vector (Table 4). Moreover, sequencing of all correctly assembled plasmids showed that the building blocks were fused in an error free manner and that no mutations were introduced during PCR.

**Table 4:** Efficiency of the versatile FAST vector assembly system.

# Building blocks	# Vectors	# Colonies min – max	Average # colonies	Cloning efficiency^a^ [%]
5	9	212–552	339	100
6	6	76–340	144	96
7	11	43–142	101	93
5+1^b^	18	21–204	52	87
Negative control^c^	10	0–10	5	-

a Cloning efficiency is based on 3 transformants for each assembled construct and calculated as the number of transformants that contained all inserts in correct orientation divided by the number of screened transformants.

b Assembly of five double-stranded PCR fragments, and one oligonucleotide-based part;

c Religation of the backbone vector fragment.

Small building blocks in the toolbox, which contain sequences that are too short to be made by PCR, were formed by annealing two partly complementary oligonucleotides. In these cases, non-homologous extensions at the 3′-ends of the two oligonucleotides provide the FAST overhangs. These building blocks include the localization sequences for endoplasmatic reticulum, mitochondria, and plasma membrane, as well as signals for retention in the medial- and trans-Golgi, and trans-Golgi network. To investigate whether these building blocks could be efficiently incorporated into vectors using the approach described above, they were mixed with five other building blocks in a number of vector assembly experiments. As a result, 18 different vectors were successfully assembled (Table 4). Compared to vector assembly, which is based solely on PCR derived building blocks, we note a minor decrease in the cloning efficiency. Even so, the number of colonies was still sufficient to achieve correctly assembled vectors in the first trial. Moreover, among the 54 vectors tested in total, 87% contained correctly fused building blocks (Table 4).

**Figure 4:**
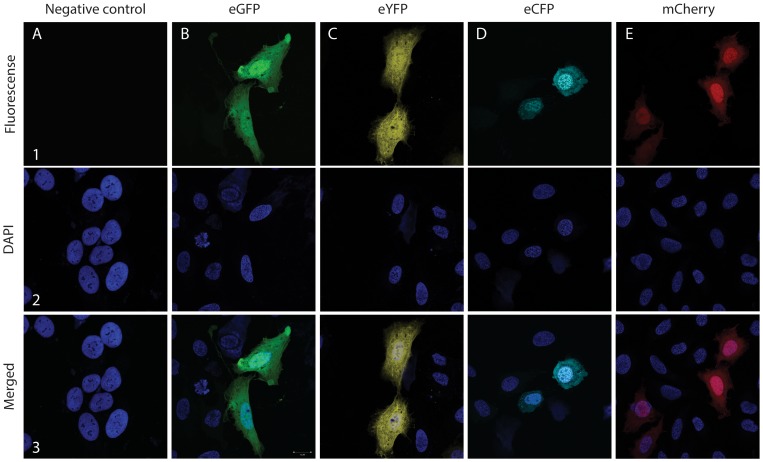
Confocal laser microscopy of fixed U-2-OS cells transiently transfected with pFAST-vectors. Confocal laser microscopy of fixed U-2-OS cells transiently transfected with control and pFAST-vectors 48h after transfection with (**A**) pC1_ccdB as negative control, (**B**) pFAST1-eGFP, (**C**) pFAST2-eYFP, (**D**) pFAST3-eCFP, and (**E**) pFAST4-mCherry. (**A1**–**E1**) microscopy with fluorescence filters. (**A2**–**E2**) nuclei stained with DAPI (dark blue). (**A3**–**E3**) merged pictures.

The vectors constructed above were examined for functionality in U-2-OS cells. Firstly, we tested vectors based on the HygR marker. After transfection, small foci of resistant cells appeared after 4 days of selection pressure. In contrast, no foci formed with cells transfected with empty vectors, see Figure S3. Secondly, we validated the functionality of building blocks forming expression cassettes for production of fluorescent proteins. Accordingly, U-2-OS cells transfected with plasmids expressing genes or gene fusions encoding eCFP, eGFP, eYFP, and mCherry were examined by fluorescent microscopy. In all cases, cells containing an easily detectable signal in the cytoplasm at the expected wavelengths were observed, see Figure 4A–E.

**Figure 5:**
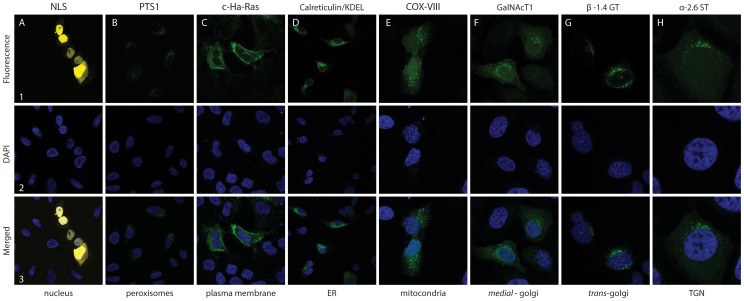
Confocal laser microscopy of U-2-OS cells expressing localized fluorescent proteins. Confocal laser microscopy of fixed U-2-OS cells transiently expressing fluorescent proteins localized to major cellular compartments. Shown are representative images of eYFP or eGFP detected 48 hours after transfection: (**A1-3**) pFAST6-eYFP::NLS, (**B1-3**) pFAST5-eGFP::PTS1, (**C1-3**) pFAST37-eGFP::c-Ha-Ras, (**D**) pFAST57-CRT::eGFP::KDEL, (**E**) pFAST58-COXVIII::eGFP, (**F**) pFAST59-GalNAcT1::eGFP, (**G**) pFAST61-b1,4GT::eGFP, (**H**) pFAST56-a-2,6ST-eGFP. (**A1**–**H1**) microscopy with fluorescence filters. (**A2**–**H2**) nuclei stained with DAPI (dark blue). (**A3**–**H3**) merged pictures.

The toolbox includes building blocks encoding cell sorting sequences containing the information to direct a protein containing no sorting signal to any of eight different locations. To test whether these building blocks could be functionally fused via FAST linkers to a GOI we made 18 new vectors, see Table S3, each encoding a fluorescent protein fused to a specific cell sorting sequence. These plasmids were transfected into U-2-OS cells and subsequently examined by fluorescent microscopy (Figure 5). In all experiments, cells containing a fluorescent signal were detected, and, as expected for functional fusions, the sorting sequences dictated the cellular locations of the tagged fluorescent proteins. For example, cells producing eYFP fused to the SV40 nuclear localization signal emitted yellow light that co-localized with DAPI stained nuclei (Figure 5A). The distribution patterns of the remaining fusion proteins (Figure 5B–H), corresponded to those already presented in the literature for proteins using these signals for sorting [17]–[27]. We therefore conclude that all protein-sorting sequences in the toolbox are functional when fused to fluorescent proteins and that the FASTs did not interfere with the localization of the targeting signal.

### Validation of protein secretion using the FAST system

Similar to intracellular targeting signals, the toolbox also includes a building block encoding a signal peptide that allows a protein to enter the secretory pathway. To test the functionality of this building block, it was fused to the gene encoding eGFP. The resulting construct and a construct coding for eGFP without the signal peptide was subsequently transfected into HEK293T cells. Transfected cells were propagated for four days and the growth medium examined for the presence of eGFP. Relative fluorescence intensity (RFI) from medium extracted from cells expressing the secreted protein was 24% and 50% higher than from medium extracted from cells expressing the non-secreted eGFP and from medium that was not inoculated with cells, respectively (Table 5). We therefore conclude that the signal peptide for secretion is functional with our FAST linker.

**Table 5:** The level of eGFP intensity in extracted media.

Plasmid	RFI
PBS	75
Blank medium	112
pFAST1-eGFP	132
pFAST55-hIFNγ::eGFP	164

RFI: relative fluorescence intensity; PBS: phosphate buffered saline.

### FAST bicistronic protein production

To investigate whether our FAST toolbox could support bicistronic gene expression, the building block containing an internal ribosomal entry site (IRES) was tested in two separate setups. Firstly, IRES was inserted between eGFP and mCherry and cells expressing this bicistronic construct were examined by fluorescence microscopy. The resulting cells contained both eGFP and mCherry in the cytoplasm. An analogous construct where the order of the two genes was reversed gave a similar result. In both cases, the dual signal cannot be the result of the formation of a fusion protein, since the two genes are not in the same reading frame, in addition, both coding sequences terminate with a stop codon. The simultaneous presence of the two fluorescent in the cytoplasm therefore strongly indicates that the ribosome was loaded at both the cap structure and at the IRES sequence of the mRNA transcribed from the plasmid. Secondly, IRES was inserted between the secreted alkaline phosphatase (SEAP) and either mCherry or eGFP. In these cases, significant extracellular activity of SEAP was detected in both experiments. Similarly, the expected fluorescent protein, but not the other, was detected in each of the two cells (Table 6, Figure S4).

**Table 6:** Amounts of SEAP in extracted media of HEK293 cells.

Plasmid	SEAP [µg/ml]
Negative control*	<0.001
pFAST53-SEAP-IRES-mCherry	0.291
pFAST54-SEAP-IRES-eGFP	0.507

SEAP: secreted alkaline phosphatase.

*Negative control was a plasmid expressing a fluorescent protein, but not SEAP.

## Conclusion

In this work, we have generated and validated a versatile vector assembly system for rapid generation of mammalian expression vectors. The system is based on FAST linker sequences and consists of two parts using this technology: the pBASE vectors allowing rapid ligation-free insertion and expression of single gene expression cassettes, and the FAST-directed assembly (pFAST vector set) allowing assembly of up to seven PCR fragments in a single cloning step. As proof of concept of the versatility, we have developed a set of constructs encoding fluorescent proteins that can be used to visualize compartments. The localization signals encoded by these constructs were 3-62 amino acids long; all were functionally fused to fluorescent proteins via our FAST linkers in the cell lines HEK293, U-2-OS, CHO-K1, and CHO-S. We have in this setup tested assembly of up to seven fragments, but the fact that these constructs were easily obtained and showed cloning efficiencies above 90%, indicates that even more complex vectors consisting of additional fragments can likely be constructed with this method. Furthermore, the FAST linkers make the system easily expandable to any components a user might wish to add. In summary, we provide a non-commercial validated method for one-step assembly of up to seven DNA fragments. The versatile vector assembly strategy we present here can therefore be adapted to a wide range of uses and broadly benefit the mammalian research community.

## Supporting Information

Figure S1 **Backbone vector preparation by digestion.** For high-throughput parallel cloning into the vector, plasmids are treated with the enzymes *PacI* and *Nt.BbvCI* to generate a vector backbone, which can be combined with genes of interest.
(TIF)

Figure S2 **Schematic representation of the flexible combination of building blocks.** The FAST-based vector assembly allows flexible combination of building blocks and therefore variants of the building block exist with different FAST. The color indicates the different FASTs.
(TIF)

Figure S3 **Characterization of functionality of the Hygromycin selectable marker in transiently transfected U-2-OS cells.** Light microscopy of U-2-OS cells transfected with control and pFAST1-eGFP. Representative images of cells are shown at day 0, 1, 4, and 8 after addition of Hygromycin B. (**A**) pC1_ccdB and (**B**) FAST1-eGFP.
(TIF)

Figure S4 **Characterization of IRES expression in transiently transfected U-2-OS cells by confocal laser microscopy.** Representative images of fluorescent expression of fixed U-2-OS are shown 48 h after transfection with **(A)** pFAST54_SEAP-IRES-eGFP, **(B)** pFAST53_SEAP-IRES-mCherry, **(C)** pFAST72_eGFP-IRES-mCherry, **(D)** pFAST73_mCherry-IRES-eGFP. Cells were fixed with paraformaldehyde, nuclei stained with DAPI, and visualized under fluorescence microscope **(A, B)** or confocal microscopy **(C, D)**.
(TIF)

Table S1 **Primer sequences.**
(DOCX)

Table S2 **Synthetic DNA oligonucleotide.**
(DOCX)

Table S3 **Plasmids constructed with the FAST assembly system of this study.**
(DOCX)
