# Supplementary figures and images for: A Versatile System for USER Cloning-Based Assembly of Expression Vectors for Mammalian Cell Engineering
Source: PLoS One. 2014 May 30;9(5):e96693. doi: 10.1371/journal.pone.0096693 (PMC4039435; doi:10.1371/journal.pone.0096693)

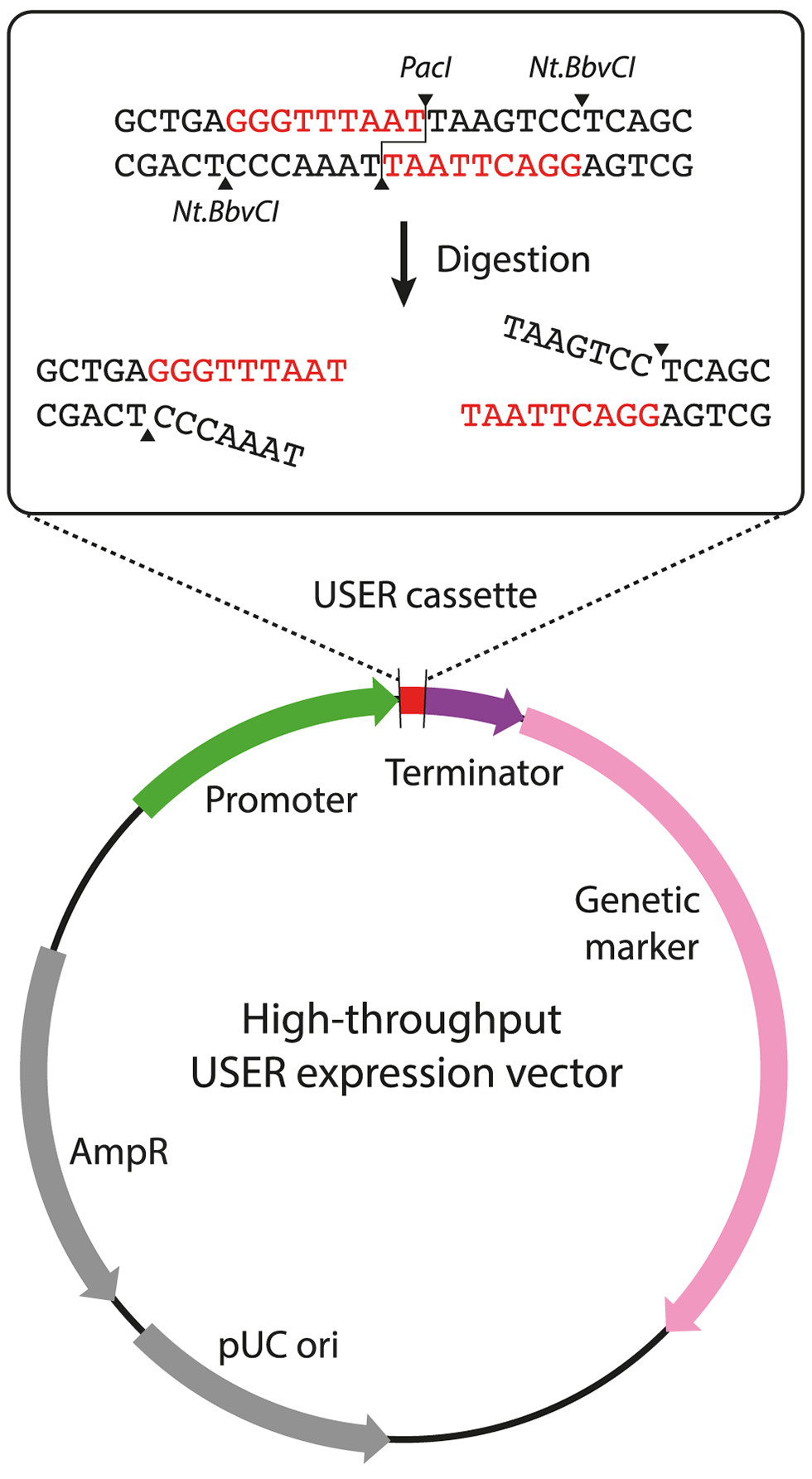

Supplement: Figure S1 — Backbone vector preparation by digestion. For high-throughput parallel cloning into the vector, plasmids are treated with the enzymes PacI and Nt.BbvCI to generate a vector backbone, which can be combined with genes of interest. (TIF) [file pone.0096693.s001.tif]

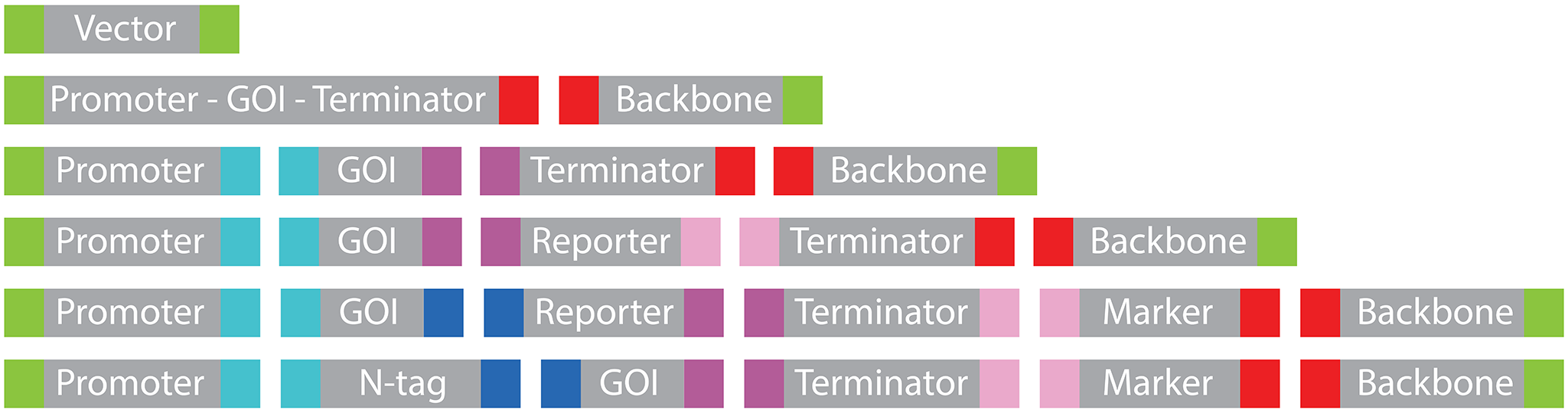

Supplement: Figure S2 — Schematic representation of the flexible combination of building blocks. The FAST-based vector assembly allows flexible combination of building blocks and therefore variants of the building block exist with different FAST. The color indicates the different FASTs. (TIF) [file pone.0096693.s002.tif]

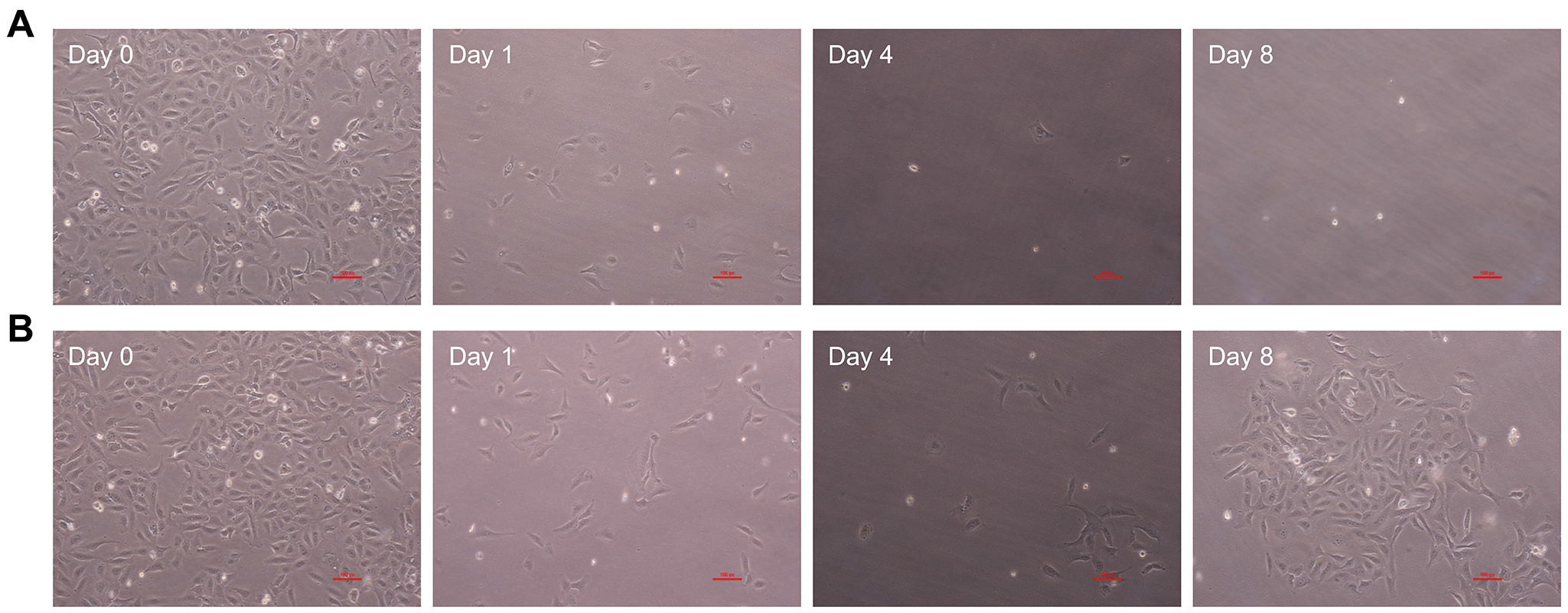

Supplement: Figure S3 — Characterization of functionality of the Hygromycin selectable marker in transiently transfected U-2-OS cells. Light microscopy of U-2-OS cells transfected with control and pFAST1-eGFP. Representative images of cells are shown at day 0, 1, 4, and 8 after addition of Hygromycin B. (A) pC1_ccdB and (B) FAST1-eGFP. (TIF) [file pone.0096693.s003.tif]

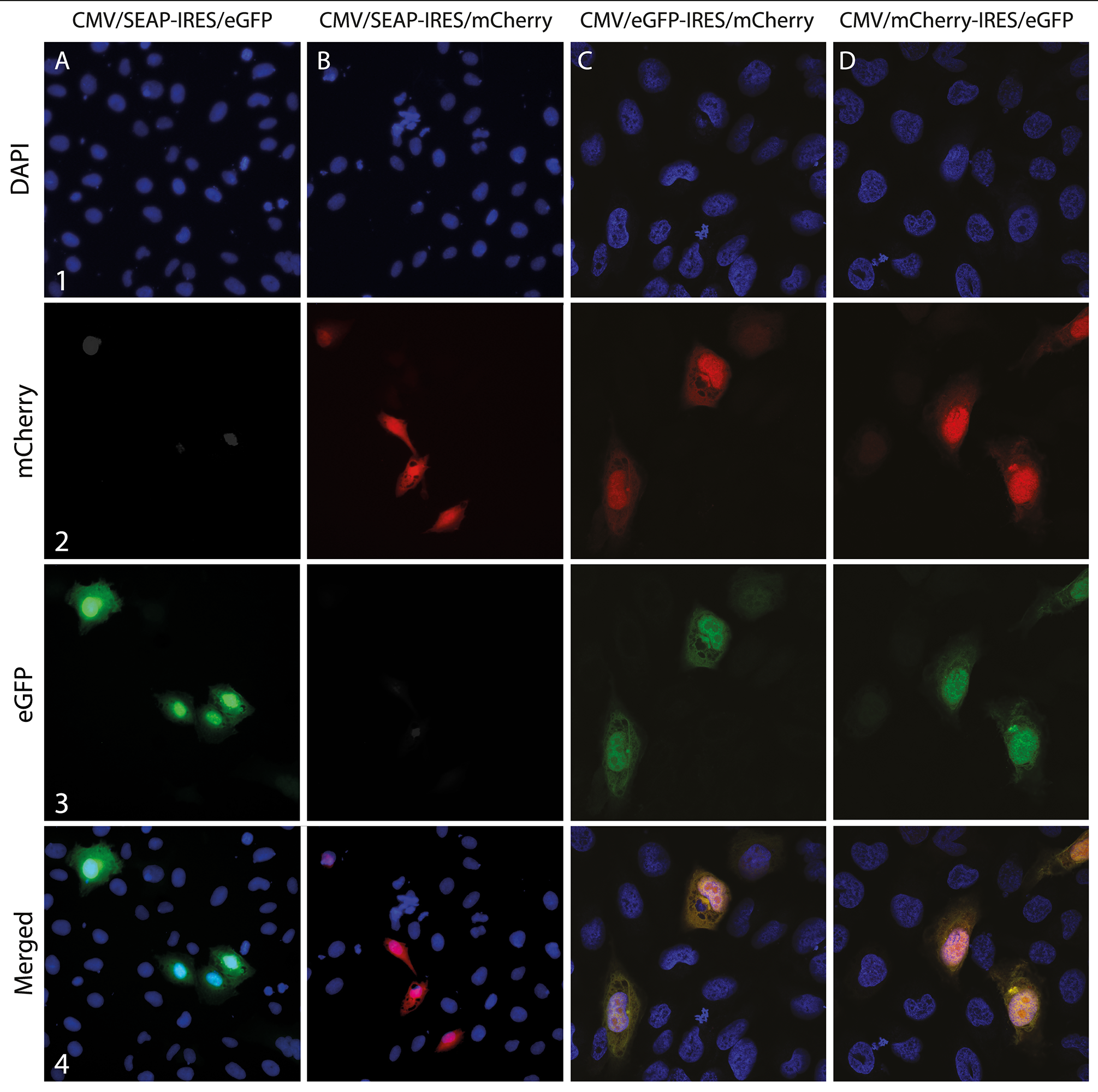

Supplement: Figure S4 — Characterization of IRES expression in transiently transfected U-2-OS cells by confocal laser microscopy. Representative images of fluorescent expression of fixed U-2-OS are shown 48 h after transfection with (A) pFAST54_SEAP-IRES-eGFP, (B) pFAST53_SEAP-IRES-mCherry, (C) pFAST72_eGFP-IRES-mCherry, (D) pFAST73_mCherry-IRES-eGFP. Cells were fixed with paraformaldehyde, nuclei stained with DAPI, and visualized under fluorescence microscope (A, B) or confocal microscopy (C, D). (TIF) [file pone.0096693.s004.tif]
