# Supplementary material for: A Versatile System for USER Cloning-Based Assembly of Expression Vectors for Mammalian Cell Engineering
Source: PLoS One. 2014 May 30;9(5):e96693. doi: 10.1371/journal.pone.0096693 (PMC4039435; doi:10.1371/journal.pone.0096693)
Supplement: Table S1 — Primer sequences. (DOCX) [file pone.0096693.s005.docx]

**Table S1. Primer sequences**

| *Name* | *Sequence (5´→ 3´)* |
| --- | --- |
| PacI-cass BGHpA(FAST2)-FW | **AGTGCGAU**GCTGAGGGTTTAATTAAGTCCTCAGCCTGTGCCTTCTAGTTGCCAGC |
| PacI-cass SV40pA(FAST2)-FW | **AGTGCGAU**GCTGAGGGTTTAATTAAGTCCTCAGCAACTTGTTTATTGCAGCTTATAATGGTTAC |
| PGK(FAST1)-FW | **ACGTCGCU**CCGGTAGGCGCCAACCG |
| PGK(FAST2)-RV | **ATCGCACU**GGCTGCAGGTCGAAAGGCC |
| SV40(FAST1)-FW | **ACGTCGCU**CTGTGGAATGTGTGTCAGTTAGG |
| SV40(FAST2)-RV | **ATCGCACU**AGCTTTTTGCAAAAGCCTAGG |
| CMV(FAST1)-FW | **ACGTCGCU**CGATGTACGGGCCAGATATAC |
| CMV(FAST2)-RV | **ATCGCACU**ATTTCGATAAGCCAGTAAGCAGT |
| SV40 pA(FAST5)-FW | **ACACAGTCU**AACTTGTTTATTGCAGCTTATAATGGTTAC |
| SV40 pA(FAST6)-RV | **ACGCAAGU**CAGACATGATAAGATACATTGATGAGTTTG |
| BGH pA(FAST5)-FW | **ACACAGTCU**CTGTGCCTTCTAGTTGCCAGC |
| BGH pA(FAST6)-RV | **ACGCAAGU**CCATAGAGCCCACCGCATC |
| hGH pA(FAST5)-FW | **ACACAGTCU**AGGATCCCGGGTGGCATC |
| hGH pA(FAST6)-RV | **ACGCAAGU**CAACAGGCATCTACTGAGTGGACC |
| Backbone(FAST7)-FW | **ATTAAGCU**AGTGAGTCGAATAAGGGCGACA |
| Backbone(FAST1)-RV | **AGCGACGU**GAGTCGAATAAGGGCGACACC |
| Kozak-FWP (PacI cass.)-FW | **GGGTTTAAU**ATGGTGAGCAAGGGCGAG |
| Kozak-FWP(FAST1)-FW | **AGTGCGAU**CGCCACCATGGTGAGCAA |
| FP(FAST2)-FW | **AGTGCGAU**ATGGTGAGCAAGGGCGAGG |
| FP(FAST3)-FW | **AGCGCTGGU**ATGGTGAGCAAGGGCGAGG |
| FP(FAST4)-FW | **ACTATGCCU**ATGGTGAGCAAGGGCGAGG |
| FP(PacI cass.)-RV | **GGACTTAAU**TTACTTGTACAGCTCGTCCATGC |
| eGFP(FAST5)-RV | **AGACTGTGU**TTACTTGTACAGCTCGTCCATGCC |
| eCFP/YFP(FAST5)-RV | **AGACTGTGU**TTATCTAGATCCGGTGGATCCC |
| mCherry(FAST5)-RV | **AGACTGTGU**CTACTTGTACAGCTCGTCCATGC |
| eGFP/mCherry(FAST3)-RV | **ACCAGCGCU**CTTGTACAGCTCGTCCATGCC |
| eYFP/CFP(FAST3)-RV | **ACCAGCGCU** TCTAGATCCGGTGGATCCCG |
| DHFR(FAST2)-FW | **AGTGCGAU**ATGGTTCGACCATTGAACTGCAT |
| DHFR(FAST5)-RV | **ATCGGAAU**TTAGTCTTTCTTCTCGTAGACTTCAA |
| SEAP(FAST2)-FW | **AGTGCGAU**ATGCTGGGGCCCTGCA |
| SEAP(FAST5)-RV | **AGACTGTGU**TCAGGGAGCAGTGGCCG |
| SEAP(FAST3)-RV | **ACCAGCGCU**TCAGGGAGCAGTGGCCG |
| IRES(FAST3)-FW | **AGCGCTGGU***AATTCCGCCCCTCTCCCCCCCCCCCCTCTCCCTCCCCCCCCCCTA*ACGTTACTGGCCGAAGCC |
| IRES(FAST4)-RV | **AGGCATAGU***TTATCA*TCGTGTTTTTCAAAGGAAAAC |
| HygR cass(FAST6)-FW | **ACTTGCGU**CCAGCAGGCAGAAGTATGCA |
| HygR cass(FAST6)-RV | **AGCTTAAU**CAGGCTTTACACTTTATGCTTCC |
| NepR cass(FAST6)-FW | **ACTTGCGU**CTGTGGAATGTGTGTCAGTTAGG |
| NeoR cass(FAST6)-RV | **AGCTTAAU**CAGACATGATAAGATACATTGATGAGTTTG |
| β-1,4GT(FAST2)-FW | **AGTGCGAU**ATGAGGCTTCGGGAGCCG |
| β-1,4GT(FAST3)-RV | **ACCAGCGCU**GCCCTGCAGCGGTGTGG |
| eGFP/mCherry-PTS1(FAST5)-RV | **AGACTGTGU**TTACAGCTTGGACTTGTACAGCTCGTCCATGCC |
| eGFP/mCherry-His tag (FAST5)-RV | **AGACTGTGU**TTAATGATGATGATGATGATGCTTGTACAGCTCGTCCATGCC |
| eGFP/mCherry-NLS(FAST5)-RV | **AGACTGTGU**TTATACCTTTCTCTTCTTTTTTGGCTTGTACAGCTCGTCCATGCC |
| eGFP/mCherry-KDEL(FAST5)-RV | **AGACTGTGU**TTACAGCTCGTCCTTCTTGTACAGCTCGTCCATGCC |

FW, forward primer; RV, reverse primer; FP, fluorescent protein; BASE, high-throughput assembly, NLS, nuclear localization sequence; PTS1, peroxisomal target signal 1. The primer sequences are shown with FAST, which FAST is indicated in the brackets. Signal sequences included in the primers are underlined. The italic nucleotides represent are the sequence added to modify in-house version of IRES to mimic the sequence of pIRES.
