# Supplementary material for: A Versatile System for USER Cloning-Based Assembly of Expression Vectors for Mammalian Cell Engineering
Source: PLoS One. 2014 May 30;9(5):e96693. doi: 10.1371/journal.pone.0096693 (PMC4039435; doi:10.1371/journal.pone.0096693)
Supplement: Table S2 — Synthetic DNA oligonucleotide. (DOCX) [file pone.0096693.s006.docx]

**Table S2. Synthetic DNA oligonucleotide**

| *Signal peptide* | *Oligonucleotide sequence reading strand (5´→ 3´)* |
| --- | --- |
| GalNAc-T1 pos (FAST3) | ATGCTGAAGAAGCAGTCTGCAGGGCTTGTGCTGTGGGGCGCTATCCTCTTTGTGGCCTGGAATGCCCTGCTGCTCCTCTTCTTCTGG**AGCGCTGGT** |
| GalNAc-T1 neg (FAST2) | CCAGAAGAAGAGGAGCAGCAGGGCATTCCAGGCCACAAAGAGGATAGCGCCCCACAGCACAAGCCCTGCAGACTGCTTCTTCAGCAT**ATCGCACT** |
| α-2,6 ST pos (FAST3) | ATGATTCACACCAACCTGAAGAAAAAGTTCAGCTACTTCATCCTGGCCTTTCTCCTGTTTGCACTCATCTGTGTGTGG**AGCGCTGGT** |
| α-2,6 ST neg (FAST2) | CCACACACAGATGAGTGCAAACAGGAGAAAGGCCAGGATGAAGTAGCTGAACTTTTTCTTCAGGTTGGTGTGAATCAT**ATCGCACT** |
| COX-VIII pos (FAST3) | ATGTCCGTCCTGACGCCGCTGCTGCTGCGGGGCTTGACAGGCTCGGCCCGGCGGCTCCCAGTGCCGCGCGCCAAGATCCATTCGTTGCCGCCG**AGCGCTGGT** |
| COX-VIII neg(FAST2) | CGGCGGCAACGAATGGATCTTGGCGCGCGGCACTGGGAGCCGCCGGGCCGAGCCTGTCAAGCCCCGCAGCAGCAGCGGCGTCAGGACGGACAT**ATCGCACT** |
| CRT pos (FAST3) | ATGCTGCTCCCTGTGCCGCTGCTGCTCGGCCTGCTCGGCCTGGCCGCCGCC**AGCGCTGGT** |
| CRT neg (FAST2) | GGCGGCGGCCAGGCCGAGCAGGCCGAGCAGCAGCGGCACAGGGAGCAGCAT**ATCGCACT** |
| IFN-γ pos (FAST3) | ATGAAATATACAAGTTATATCTTGGCTTTTCAGCTCTGCATCGTTTTGGGTTCTCTTGGC**AGCGCTGGT** |
| IFN-γ neg (FAST2) | GCCAAGAGAACCCAAAACGATGCAGAGCTGAAAAGCCAAGATATAACTTGTATATTTCAT**ATCGCACT** |
| c-Ha-ras pos (FAST5) | CTGAACCCTCCTGATGAGAGTGGCCCCGGCTGCATGAGCTGCAAGTGTGTGCTCTCCTGATAA**ACACAGTCT** |
| c-Ha-ras neg (FAST3) | TTATCAGGAGAGCACACACTTGCAGCTCATGCAGCCGGGGCCACTCTCATCAGGAGGGTTCAG**ACCAGCGCT** |
| β-1,4 GT_1 | ATGAGGCTTCGGGAGCCGCTCCTGAGCGGCAGCGCCGCGATGCCAGGCGCGTCCCTACAGCGGGCCTGCCGCCTGCTCGTGGCCGTCTGCGCTCTGCACCTTGG |
| β-1,4 GT_2 | GCCCTGCAGCGGTGTGGAGACTCCGACCAGTTGGGGCAGGCGGCTCAGGTCGCGGCCAGCCAGGTAGTAAACGAGGGTGACGCCAAGGTGCAGAGCGCAGAC |
| The sequences are shown with FAST, which FAST is indicated in the brackets. GalNAcT1, N-terminal targeting signal of N-acetylgalactosaminyltransferase; α-2,6 ST, N-terminal targeting signal of beta-galactoside alpha-2,6-sialyltransferase; COX-VIII, N-terminal targeting signal of cytochrome c oxidase subunit VIII; CRT, N-terminal targeting signal of calreticulin; IFN-γ, N-terminal targeting signal of interferon-gamma; c-Ha-ras, C-terminal targeting signal of c-Ha-ras p21 protein ; β-1,4 GT, N-terminal targeting signal of beta-1,4-galactosyltransferase; pos, positive strand; neg, negative strand. | |
