# Supplementary material for: A Versatile System for USER Cloning-Based Assembly of Expression Vectors for Mammalian Cell Engineering
Source: PLoS One. 2014 May 30;9(5):e96693. doi: 10.1371/journal.pone.0096693 (PMC4039435; doi:10.1371/journal.pone.0096693)
Supplement: Table S3 — Plasmids constructed with the FAST assembly system of this study. (DOCX) [file pone.0096693.s007.docx]

**Table S3. Plasmids constructed with the FAST assembly system of this study.**

| *Plasmid name* | *Relevant characteristics* | *# Building blocks* |
| --- | --- | --- |
| pBASE1 | CMV/PacI-Nt.BbvCI_BGHpA_HygR | 3 |
| pBASE2 | CMV/PacI-Nt.BbvCI_BGHpA _NeoR | 3 |
| pBASE3 | SV40/PacI-Nt.BbvCI_SV40pA HygR | 3 |
| pBASE4 | SV40/PacI-Nt.BbvCI_SV40pA_NeoR | 3 |
| pBASE5 | PGK/PacI-Nt.BbvCI_BGHpA_HygR | 3 |
| pBASE6 | PGK/PacI-Nt.BbvCI_BGHpA_NeoR | 3 |
| pBASE2-eGFP | CMV/kz-eGFP/cyto_BGHpA_NeoR | 1 + pBASE |
| pC1-eGFP | CMV/kz-eGFP/cyto_BGHpA | 1 + pC1_ccdB |
| pFAST1-eGFP | CMV/kz-eGFP/cyto_BGHpA_HygR | 5 |
| pFAST2-eYFP | CMV/kz-eYFP/cyto_BGHpA_HygR | 5 |
| pFAST3-eCFP | CMV/kz-eCFP/cyto_BGHpA_HygR | 5 |
| pFAST4-mCherry | CMV/kz-mCherry/cyto_BGHpA_HygR | 5 |
| pFAST5-eGFP-PTS1 | CMV/kz-eGFP-PTS1/perox_BGHpA _HygR | 5 |
| pFAST6-eYFP-NLS | CMV/kz-eYFP-NLS/nucleus_BGHpA_HygR | 5 |
| pFAST25-mCherry-NLS | CMV/kz-mCherry-NLS/nucleus_BGHpA_HygR | 5 |
| pFAST32 | CMV/kz-eGFP-PTS1/perox-IRES/kz-mCherry-NLS /nucleus_BGHpA_HygR | 7 |
| pFAST33 | CMV/kz-eGFP-NLS/nucleus_IRES/kz-eGFP-PTS1/perox_BGHpA_HygR | 7 |
| pFAST37 | pCMV/kz-eGFP-c-Ha-ras/mem_BGHpA_HygR | 6 |
| pFAST38 | pCMV/kz-eYFP-c-Ha-ras/mem_BGHpA _HygR | 6 |
| pFAST39 | pCMV/kz-mCherry-c-Ha-ras/mem_BGHpA _HygR | 6 |
| pFAST54 | CMV/SEAP-IRES/eGFP/cyto_BGHpA_NeoR | 7 |
| pFAST55 | CMV/IFN-y-eGFP/sec_BGHpA_HygR | 6 |
| pFAST56 | CMV/α-2,6ST-eGFP/TGN_BGHpA_HygR | 6 |
| pFAST57 | CMV/CRT-eGFP-KDEL/ER_BGHpA_HygR | 6 |
| pFAST58 | CMV/COXVIII-eGFP/mito_BGHpA_HygR | 6 |
| pFAST59 | CMV/GalNAcT1-eGFP/*medial*-golgi_BGHpA_HygR | 6 |
| pFAST60 | CMV/IFN-y-eGFP/His-tag/sec_BGHpA_HygR | 6 |
| pFAST61 | CMV/β-1,4GT-eGFP/*trans*-golgi_BGHpA_HygR | 6 |
| pFAST62 | CMV/β-1,4GT-mCherry/*trans*-golgi_BGHpA _HygR | 6 |
| pFAST63 | CMV/α-2,6ST-eYFP/TGN_BGHpA_HygR | 6 |
| pFAST64 | CMV/eCFP-GalNAcT1/*medial*-golgi_BGHpA_HygR | 6 |
| pFAST66 | CMV/CRT-eGFP-KDEL/ER-mCherry-NLS /nucleus_BGHpA _HygR | 6 |
| pFAST67 | CMV/mCherry-NLS/nucleus-CRT-eGFP-KDEL/ER_BGHpA_HygR | 6 |
| pFAST68 | CMV/ CRT-eGFP-KDEL/ER-IRES/mCherry-NLS/nucleus_BGHpA_HygR | 7 |
| pFAST69 | CMV/mCherry-NLS/nucleus-IRES/ CRT-eGFP-KDEL/ER_BGHpA _HygR | 7 |
| pFAST72 | CMV/mCherry-IRES/eGFP_BGHpA _HygR | 7 |
| pFAST73 | CMV/eGFP-IRES/mCherry_BGHpA _HygR | 7 |
| pFAST_SEAP_1 | CMV/SEAP_BGHpA _NeoR | 5 |
| pFAST_SEAP-DHFR | CMV/SEAP_BGHpA _DHFR | 6 |
| pFAST-DHFR | SV40/DHFR_SV40pA | 3 |
| pFAST-Neo | SV40/ NeoR_SV40pA | 3 |
| pFAST-Hyg | SV40/ HygR_SV40pA | 3 |

kz, kozak sequence; cyto, cytoplasmic expression; NLS, neulear localization signal; PTS1, peroxisomal targeting signal 1; perox, peroxisomal expression; mito, mitochondrial expression; mem, membrane bound expression; sec, secretion; TGN, trans-golgi network; CRT, calreticulin signal peptide; KDEL, ER retention signal; ER, endoplasmic reticulum.
